# Diagnostic accuracy of arthroscopic biopsy in periprosthetic infections of the hip

**DOI:** 10.1186/s40001-017-0246-0

**Published:** 2017-03-04

**Authors:** Florian Pohlig, Heinrich M. L. Mühlhofer, Ulrich Lenze, Florian W. Lenze, Christian Suren, Norbert Harrasser, Rüdiger von Eisenhart-Rothe, Johannes Schauwecker

**Affiliations:** 1 Department of Orthopedic Surgery, Klinikum rechts der Isar, Technical University Munich, Ismaninger Str. 22, 81675 Munich, Germany; 2 Department of Traumatology, Klinikum Traunstein, Cuno-Niggl-Str. 3, 83278 Traunstein, Germany

**Keywords:** Hip arthroscopy, Periprosthetic infection, Biopsy, Aspiration, PJI, Revision hip arthroplasty

## Abstract

**Background:**

Diagnosis of a low-grade periprosthetic joint infection (PJI) prior to revision surgery can be challenging, despite paramount importance for further treatment. Arthroscopic biopsy of synovial and periprosthetic tissue with subsequent microbiological and histological examination can be beneficial but its specific diagnostic value has not been clearly defined.

**Methods:**

20 consecutive patients who underwent percutaneous synovial fluid aspiration as well as arthroscopic biopsy due to suspected PJI of the hip and subsequent one- or two-stage revision surgery at our institution between January 2012 and May 2015 were enrolled. Indication was based on the criteria (1) history of PJI and increased levels of erythrocyte sedimentation rate (ESR) or C-reactive protein (CRP), (2) suspicious cell count and differential but negative bacterial culture in synovial aspirate, (3) early loosening (<less than 2 years), or (4) persisting pain without loosening but history of a PJI. At least two criteria had to be fulfilled in order to perform an arthroscopic biopsy.

**Results:**

Best overall diagnostic value was identified for arthroscopic biopsy and a combination of bacteriological and histological analysis with a sensitivity of 87.5%, specificity of 100% and accuracy of 95%. Bacteriological assessment of synovial aspirate revealed a sensitivity of 50.0%, specificity of 91.7%, and accuracy of 75%. ESR and CRP yielded a sensitivity of 75.0% for either hematologic test and specificities of 87.5 and 66.7%, respectively.

**Conclusions:**

In conclusion, our data indicate that arthroscopic biopsy is superior to ESR and CRP as well as joint aspiration and their combinations. Concurrent microbiological and histological examination of the biopsy specimens allows for identification of the causative pathogen and its susceptibility pattern in order to preoperatively plan the surgical strategy as well as the antibiotic regimen. Moreover, intraarticular mechanical failure can be detected during hip arthroscopy emphasizing its diagnostic value. Level II diagnostic study.

## Background

Osteoarthritis (OA) of the hip is a disabling condition with significant and rising incidences due to the demographic development in modern industrial countries. Among different symptomatic treatment options, total hip arthroplasty (THA) remains the only causal therapy. Thus, the number of THA performed in the USA is estimated to triple until 2030 leading to a significant increase of revision surgery [1].

One major reason for revision arthroplasty is periprosthetic joint infection (PJI). Recent literature suggests that revision surgery of THA is performed in up to 15% of the cases due to PJI [2]. In contrast to acute PJIs, low-grade infections are frequently caused by low-virulent bacterial strains of the skin flora, e.g., *coagulase*-*negative staphylococci (CNS)*, often lacking severe inflammatory symptoms. Despite paramount importance for further treatment, diagnosis of a low-grade PJI prior to revision surgery can be challenging.

In addition to clinical findings, erythrocyte sedimentation rate (ESR), C-reactive protein (CRP), and percutaneous aspiration of synovial fluid for evaluation of cell count and differential as well as microbiological analysis depict routinely employed diagnostic tools [3]. These tests have an important role in the workup of a tentative PJI; however, diagnostic values vary greatly in recent literature [4–6]. Despite Johnson et al. reporting sensitivities of 91% for ESR and 95% for CRP, exclusive use of hematologic tests to rule out PJI can be delusive due to a considerable subset of patients with present PJI and negative serology [4].

Similarly, for percutaneous aspiration of synovial fluid sensitivity varies from 12 to 89% and specificity from 50 to 100% (Table 1) [6–17]. Although identification of the causative pathogen and its antibiotic susceptibility pattern is essential for a suitable treatment strategy, its overall diagnostic accuracy is still not satisfactory [18].

**Table 1:** Diagnostic value of microbiological culture of synovial fluid aspirate for diagnosis of PJI of the hip

Study	Year	Number of hips	Sensitivity (%)	Specificity (%)	PPV (%)	NPV (%)	Accuracy (%)
Ali et al. [7]	2006	73	82	91	74	94	89
Barrack & Harris [8]	1993	260	50	88	6	99	87
Cross et al. [9]	2014	110	59	100	100	93	94
Fehring & Cohen [10]	1996	166	50	88	50	89	87
Itasaka et al. [11]	2001	29	40	92	50	88	83
Kraemer et al. [12]	1993	45	57	97	89	83	84
Lachiewicz et al. [13]	1996	128	85	97	85	97	95
Müller et al. [14]	2008	50	57	50	78	29	54
Somme et al. [15]	2003	109	83	100	100	86	92
Spangehl et al. [6]	1999	202	86	94	67	98	93
Steinbrink & Frommelt [16]	1995	2158	82	96	87	94	92
Williams et al. [17]	2004	273	80	94	81	93	90

In order to improve the diagnostic yield in suspected PJI of the hip, some authors propose tissue biopsies for microbiological and histological analyses. In a prospective study, Fink et al. report a superior sensitivity of 82% and specificity of 98% for percutaneous fluoroscopically controlled tissue biopsy compared to 64 and 96% for sole aspiration of the index hip joint, respectively [5]. In contrast, Cross and colleagues identified an inferior sensitivity of synovial biopsy utilizing a fine-needle technique of 41% compared to 59% for aspiration and a specificity of 100% for either procedure [9].

We therefore (1) compared the diagnostic yield of ESR and CRP, cell count and differential of synovial aspirate, microbiological culture of aspirate and biopsy samples, histological analysis of biopsy specimens as well as reasonable combinations of these diagnostic tools and (2) hypothesized that arthroscopically controlled tissue biopsy with subsequent microbiological and histological analyses exhibits superior results. To the best of our knowledge, it is the first prospective study concerning the diagnostic value of hip arthroscopy in tentative PJI.

## Patients and methods

### Patients

All patients who underwent percutaneous synovial fluid aspiration as well as arthroscopic biopsy due to suspected PJI of the hip and subsequent one- or two-stage revision surgery at our institution between January 2012 and May 2015 were enrolled.

### Indication

Indication for arthroscopic biopsy prior to arthroplasty revision surgery was based on the tentative diagnosis of a PJI. According to our own evidence-based diagnostic algorithm, an aspiration without anesthesia was performed in all patients prior to inclusion in our study. In case of obvious infections, for example pus in the aspirate or presence of a fistula, patients were excluded. Only patients with negative or unclear results from an aspiration without anesthesia and the presence of at least 2 of the following 4 criteria were enrolled: (1) history (delayed wound healing, postoperative superficial wound healing, persisting wound drainage, pain) and increased CRP (greater than 0.5 mg/dl) or ESR (greater than 30 mm/h), (2) conspicuous cell count and differential in synovial aspirate, (3) early loosening (<less than 2 years), or (4) persisting pain without loosening but history of a PJI. Antibiotic treatment, if applicable, was terminated at least 2 weeks prior to the intervention.

### Aspiration and arthroscopic biopsy

Percutaneous aspiration and arthroscopic biopsy were performed in the operating room under strictly aseptic conditions and general anesthesia. The patient was placed supine on a radiolucent table. After preoperative skin preparation with antiseptic agent (iodine) and sterile covering, a small skin incision for a standard anterolateral arthroscopy portal was made. Under fluoroscopic control, a 14-gauge spinal needle was placed within the joint and at least 4 ml of synovial fluid was attained. The aspirate was divided for cell count and differential analysis as well as bacterial cultures. For the latter, the aspirate was transferred into anaerobe and aerobe blood culture flasks.

Subsequently, an anterolateral and lateral arthroscopy portal were established. Prior to instillation of arthroscopy fluid, 5 specimens from different locations of the periprosthetic space and the synovia were obtained under direct optical control. All biopsy samples were divided for histological analysis and microbiological cultures. After instillation of arthroscopy fluid, a diagnostic exploration for wear disease or mechanical reasons of prosthetic failure completed the arthroscopy.

### Revision surgery

Based on the results of the biopsy, one- or two-stage arthroplasty revision surgery was performed. Intraoperatively, at least 5 samples including synovial fluid, synovial tissue, and periprosthetic membrane were obtained. Again, all samples were divided and subjected to bacteriological and histological analysis. Perioperative antibiotics were withheld until retrieval of all tissue samples. The combined microbiological and histological results of the specimens obtained during revision surgery as well as sonication of the removed implant were used as definitive diagnostic test.

### Microbiology and histology

All samples for microbiologic analysis were routinely inoculated for 10 days. In case of any suspicion of growth, samples were subcultured in a thioglycollate broth for another 4 days. A result was considered positive if at least two cultures showed growth of the same pathogen. Isolated growth in only one culture was considered as contaminant and thus as negative result except for concurrent positive histologic findings.

Specimens for histological workup were routinely processed and stained using periodic acid-Schiff (PAS) and CD15 immunohistochemistry. During further analysis, neutrophilic granulocytes in the histologic sections were enumerated. A threshold of ≥23 neutrophilic granulocytes in 10 high power fields (HPF) was considered as PJI as previously recommended by Morawietz et al. [19].

Final diagnosis in terms of the presence or absence of a PJI was made upon discussion in our “Endoprosthetic Infection Board” together with microbiologists and pathologists specialized in periprosthetic infections.

### Statistics

All diagnostic parameters, ESR, CRP, cell count and differential of synovial aspirate, microbiologic culture of aspirate and biopsy samples, histologic results of biopsy specimens as well as any reasonable combination were compared to the definitive results obtained during arthroplasty revision surgery. Sensitivity, specificity, positive predictive value (PPV), negative predictive value (NPV), diagnostic accuracy (true positives and true negatives divided by the total number of patients), positive and negative likelihood ratios (PLR; NLR) as well as 95% confidence intervals were calculated. ESR, CRP, and cell count values were compared using SPSS Software (IBM, Armonk, NY, USA) and the Mann–Whitney-*U* test for independent samples. A *p* value ≤0.05 was considered statistically significant. Following Rothman, no Bonferoni adjustment for multiple testing was performed due to the observational nature of our data [20].

## Results

Best overall diagnostic value of all procedures investigated in this study for identification of PJI was achieved by arthroscopic biopsy and a combination of bacteriological and histological analysis of the specimens (Table 2). Adjacent to arthroscopic biopsy, combined examination of ESR, CRP, and cell count as well as neutrophil percentage from synovial fluid aspirate and additional microbiological assessment yielded the highest overall diagnostic value as shown in Table 2. Hereafter, slightly inferior results were obtained for a concurrent evaluation of ESR and cell count/neutrophil percentage followed by the combined assessment of CRP values and synovial aspirate (Table 2).

**Table 2:** Diagnostic value of different diagnostic tools and their combinations for diagnosis of PJI of the hip

	Aspiration (microbiology)	Aspiration (cell count/percentage neutrophiles)	Arthroscop. biopsy (microbiology)	Arthroscop. biopsy (microbiology + histology)	ESR	CRP	Aspiration (ESR + cell count/percentage neutrophiles)	Aspiration (CRP + cell count/percentage neutrophiles)	Aspiration (ESR + CRP + cell count/percentage neutrophiles + microbiology)
Sensitivity (95% CI in %)	50.0% (15.7–84.3)	80.0% (28.4–99.5)	75.0% (34.9–96.8)	87.5% (47.7–99.7)	75.0% (19.4–99.4)	75.0% (34.9–96.8)	75.0% (19.4–99.4)	80.0% (28.4–99.5)	87.5% (47.7–99.7)
Specificity (95% CI in %)	91.7% (61.5–99.8)	60.0% (14.7–94.7)	83.3% (51.6–97.9)	100% (73,5–100)	87.5% (47.4–99.7)	66.7% (34.9–90.1)	100% (29.2–100)	80,0% (28.4–99.5)	91.7% (61.5–99.8)
PPV (95% CI in %)	80.0% (28.4–99.5)	66.7% (22.3–95.7)	75.0% (44.3–91.9)	100% (59.0–100)	75.0% (19.4–99.4)	60.0% (26.2–87.8)	100% (29.2–100)	80.0% (28.4–99.5)	87.5% (47.7–99.7)
NPV (95% CI in %)	73.3% (44.9–92.2)	75.0% (19.4–99.4)	83.3 (59.5–94.5)	92% (64.0–99.8)	87.5% (47.4–99.7)	80.0% (44.4–97.5)	75.0% (19.4–99.4)	80.0% (28.4–99.5)	91.7% (61.5–99.8)
LR+ (95% CI)	6.00 (0.8–44.4)	2.00 (0.6–6.4)	4.50 (1.2–17.0)	21.70 (1.4–333.4)	6.00 (0.9–40.9)	2.25 (0.9–5.5)	5.60 (0.4–79.7)	4.00 (0.7–24.4)	10.50 (1.6–69.8)
LR− (95% CI)	0.55 (0.3–1.1)	0.33 (0.05–2.2)	0.30 (0.09–1.02)	0.17 (0.04–0.8)	0.29 (0.05–1.6)	0.38 (0.1–1.3)	0.34 (0.09–1.4)	0.25 (0.04–1.5)	0.14 (0.02–0.9)
Accuracy	75%	70%	80%	95%	83%	70%	86%	80%	90%

Individual analysis of each diagnostic test revealed a significantly increased ESR per hour of 39.75 ± 12.55 mm in patients with verified PJI according to revision surgery compared to non-infected hips (15.50 ± 9.41; *p* = 0.008). Similar to ESR, in patients with present PJI, a significantly higher CRP-value of 1.91 ± 1.54 mg/dl compared to non-infected hip joints (0.51 ± 0.33 mg/dl; *p* = 0.031) was observed. However, 25% of hips with PJI showed normal CRP values of 0.5 mg/dl or less. Similar results were obtained for percutaneous aspiration of synovial fluid and subsequent analysis of cell count and differential as shown in Table 2. Furthermore, patients with PJI showed significantly higher cell counts in synovial fluid aspirates compared to non-infected hip joints (14.8 ± 10.7 G/l vs. 0.9 ± 1.2 G/l; *p* = 0.032). Likewise, bacteriological assessment of synovial aspirate or arthroscopic biopsy specimens revealed similar diagnostic accuracies (Table 2).

**Fig. 1:**
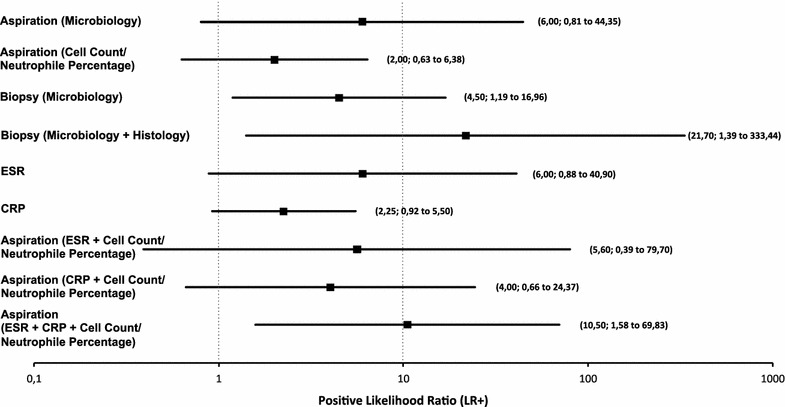
Forrest plot of positive likelihood ratios (LR+) of all examined tools and their combinations for diagnosing PJI of the hip. *Squares mark* the LR+ values whereas the horizontal lines indicate the 95% confidence interval. Values of >10 exhibit a high probability of a patient with PJI having a positive test result (compare Table 3)

In addition, likelihood ratios as criteria for the quality of a diagnostic test as suggested by Jaescke et al. were compared (Table 3) [21]. We found the highest PLR, as indicator for the presence of a PJI, for the combined microbiological and histological evaluation of arthroscopic biopsy specimens (21.7; 95% CI 1.4–333.4) followed by synovial aspirate with sole microbiologic workup (6.0; 95% CI 0.8–44.4) and concurrent assessment of ESR, CRP, cell count as well as differential and microbiological analysis from synovial aspirate (10.5; 95% CI 1.6–69.8) (Table 2; Fig. 1). Sole examination of cell count and neutrophil percentage in aspirate or analysis of CRP values yielded the lowest PLR with 2.0 (95% CI 0.6–6.4) and 2.3 (95% CI 0.9–5.5), respectively (Table 2; Fig. 1).

**Table 3:** Positive (LR+) and negative (LR−) likelihood ratios and their corresponding effect on posttest probability according to Jaeschke et al. [21]

LR+	LR−	Effect on posttest probability
>10	<0.1	Large
5–10	0.1–0.2	Moderate
2–5	0.2–0.5	Small
1–2	0.5–1	Marginal
1	1	No change

In total, 8 out of 20 cases were classified as PJI according to revision surgery resulting in a prevalence of 40%. Overall, 4 different microorganisms were identified (*Staphylococcus epidermidis, Staphylococcus capitis, Streptococcus ovis, Enterococcus faecalis)*, whereupon *Staphylococcus epidermidis* was found in 62.5%. Inoculation time until a culture was found positive varied depending on the bacterial strain and is shown in Table 4. During arthroscopy, intraarticular mechanical failure was identified in 4 patients comprising anterior impingement and wear disease in 2 cases, respectively. No complications associated with the arthroscopic procedure were observed in this study.

**Table 4:** Microorganisms identified in PJI of the hip and their time and frequency of detection

Microorganism	Number of infected joints	Time to positive culture			
		24 h	48 h	10 days	14 days
*Staphylococcus epidermidis*	5			3	2
*Staphylococcus capitis*	1			1	
*Streptococcus ovis*	1		1		
*Enterococcus faecalis*	1	1			
Total	8	1	1	4	2

## Discussion

Diagnosis of low-grade PJI frequently caused by low-virulent bacterial strains of the skin flora prior to revision surgery can be challenging. However, the accuracy of preoperative diagnosis is of fundamental importance because treatment strategies differ greatly between septic and aseptic revision surgery with expansive consequences for the patient [18].

Among routinely applied diagnostic tools, ESR and CRP are widely available, non-invasive and cost effective. In our study, we found a sensitivity of 75% and specificity of 87.5% for ESR as well as a sensitivity of 75% and specificity of 66.7% for CRP. Although sensitivities of over 90% have been published for ESR and CRP, in a recent metaanalysis Berbari et al. report pooled sensitivities of 75 and 80% as well as specificities of 70 and 74%, respectively, confirming our results [22]. However, exclusive use of hematologic tests can be delusive as McArthur et al. identified a relevant subset of patients with negative serology within their series of 414 infected THAs accounting for false negative results and thus a decreased sensitivity [4]. On the other hand, specificity might be influenced by the inclusion of patients with rheumatoid arthritis. These patients often exhibit increased CRP levels in the absence of a PJI leading to false positive results [23, 24].

Many authors propose routine joint aspiration prior to any arthroplasty revision surgery even in the absence of increased ESR and CRP levels or history of PJI [6, 15, 17, 25]. In contrast, AAOS guidelines recommend percutaneous aspiration of synovial fluid and subsequent examination of cell count and differential as well as bacteriological workup only in cases of suspected PJI [3]. If a PJI is present, however, joint aspiration can be beneficial in order to identify the causative pathogen and preoperatively plan the surgical strategy as well as the antibiotic regimen. In our study, we identified comparable results to other studies calculating to a sensitivity of 50%, specificity of 91.7%, PPV of 80%, NPV of 73%, and accuracy of 75% (Tables 1, 2). Insufficient incubation time of less than 10 days may yield poor results as Johnson et al. and Teller et al. published a sensitivity of 12 and 28%, respectively [26, 27]. In the present study, a combination of ESR, CRP, and joint aspiration yielded a sensitivity of 87.5%, specificity of 91.7%, and accuracy of 90% providing an advantage over exclusive use of either diagnostic test. Similar results were published by Fink and colleagues reporting a sensitivity of 84%, specificity of 87%, and accuracy of 86% for combined evaluation of CRP, aspiration, and biopsy [5].

Greatest diagnostic value in our study was observed for concurrent microbiological and histological examination of arthroscopically obtained biopsy specimens calculating to a sensitivity of 87.5% and specificity of 100%. In contrast to our results, Cross et al. report an inferior sensitivity of 41% of synovial biopsy utilizing a fine-needle biopsy technique and local anesthesia compared to sole percutaneous joint aspiration for diagnosing PJI in a retrospective study of 110 THAs [9]. Likewise, Williams and colleagues could not identify beneficial results for tissue biopsy and sole bacteriological examination [17]. They report a sensitivity of 83% and specificity of 90% for tissue biopsy compared to 80 and 94% for sole aspiration of the index hip joint, respectively [17]. Confirming the importance of additional histological examination of biopsy samples, Malhotra and Morgan demonstrated superior results of core needle biopsy with concurrent microbiological and histological analyses in a retrospective study of 41 hip joints [28]. The procedure necessitated general anesthesia and thus diminished the advantage of fine-needle biopsy in local anesthesia in an outpatient setting. The authors report a sensitivity of 80%, specificity of 100%, and accuracy of 97% for synovial biopsy compared to a sensitivity of 44%, specificity of 91%, and accuracy of 80% for synovial fluid aspiration [28]. Similarly, in a recent study by Fink et al., synovial biopsy was performed with arthroscopic biopsy forceps and under fluoroscopic guidance [5]. They calculated a sensitivity of 87%, specificity of 98%, and accuracy of 93%. Interestingly, the authors identified slightly inferior results for synovial biopsy of the hip compared to a previous study of the same group regarding diagnosis of PJI in total knee arthroplasty [29]. The underlying hypothesis for this discrepancy was that biopsy samples can be obtained at many more places adjacent to the prosthesis in the knee compared to hip joints, where only the head and neck of the prosthesis as well as the inlay of the acetabular cup are easily accessible [5]. This assumption would suggest superior results for synovial biopsy samples obtained from the periprosthetic membrane under direct optical control, as available during hip arthroscopy. However, our results are similar to those published by Fink et al. questioning, at least to some extent, their hypothesis [5].

Moreover, during hip arthroscopy, we could identify intraarticular mechanical failure including wear disease and anterior impingement in 20% of our cases representing diagnostic challenges as previously suggested by Pattyn et al. [30]. These results indicate a benefit of hip arthroscopy over fluoroscopically guided biopsy as published by Fink et al. since general anesthesia is required for either procedure and no complications associated with the arthroscopic intervention were observed in our study.

In conclusion, our data indicate that arthroscopic biopsy is superior to ESR and CRP as well as joint aspiration and their combinations. Concurrent microbiologic and histologic examination of the biopsy specimens allow for identification of the causative pathogen and its sensitivity pattern in order to preoperatively plan the surgical strategy as well as the antibiotic regimen. Moreover, intraarticular mechanical failure can be detected during hip arthroscopy emphasizing its diagnostic value. Indication for arthroscopic biopsy, however, should be carefully considered and based on history of PJI, clinical findings, radiographs, increased levels of ESR and CRP, and conspicuous joint aspirate.

## Abbreviations

OA: osteoarthritis
THA: total hip arthroplasty
ESR: erythrocyte sedimentation rate
CRP: C-reactive protein
PPV: positive predictive value
NPV: negative predictive value
PLR: positive likelihood ratio
NLR: negative likelihood ratio
